# Interference of kallikrein 1b26 (klk1b26) translation by microRNA specifically expressed in female mouse submandibular glands: an additional mechanism for sexual dimorphism of klk1b26 protein in the glands

**DOI:** 10.1186/2042-6410-2-13

**Published:** 2011-11-16

**Authors:** Kinji Kurihara, Nobuo Nakanishi, Akito Tomomura

**Affiliations:** 1 Department of Physiology, Meikai University School of Dentistry, Sakado, Saitama 350-0283, Japan; 2 Department of Biochemistry, Meikai University School of Dentistry, Sakado, Saitama 350-0283, Japan

**Keywords:** kallikrein, klk1b26, microRNA, salivary gland, sex difference, testosterone, translation

## Abstract

**Background:**

Mouse kallikrein 1b26 (klk1b26) protein is more abundant in male submandibular glands (SMGs) than in female ones. This sexual dimorphism has been thought to be due to increased mRNA synthesis stimulated by androgen. However, the klk1b26 protein level in female SMG is far less than that expected from the mRNA level, suggesting an additional mechanism for down-regulation of klk1b26 expression in female SMGs.

**Methods:**

We examined the effects of small non-coding RNAs in mouse SMGs on *in vitro *translation of klk1b26 using a reticulocyte lysate system and reverse transcription (RT)-PCR for klk1b26 mRNA. Statistical analyses were performed with a computer package (Microsoft Excel).

**Results:**

The microRNA (miRNA) preparation from female SMGs, but not male SMGs, interfered with the *in vitro *translation of the klk1b26 protein and inhibited the RT-PCR for klk1b26 mRNA with forward primers targeting its 5'-terminal region (between the 15th and 40th nucleotide from the 5'-terminal). The miRNA preparation from castrated mouse SMGs showed the inhibitory effect on the klk1b26 translation, but that from a 5α-dihydrotestosterone-treated female mouse SMGs did not. Synthetic miRNAs (miR-325 and miR-1497a), which have partial complementarity with klk1b26 mRNA at its 5'-terminal region (15th to 40th nucleotide position from the 5'-terminal), also interfered with the *in vitro *klk1b26 translation. When the female miRNA preparation was incubated with a 30-nucleotide-long single-strand oligoDNA (named [15th-44th]ssDNA, whose sequence corresponded to the 15th to 44th position from the 5'-terminal of klk1b26 mRNA) prior to the addition into the *in vitro *translation system, the inhibitory effect of the miRNA preparation on klk1b26 translation disappeared, while [15th-44th]ssDNA itself had no effect on the translation. Preincubation of the miRNA preparation with another single-strand DNA ([169th-198th]ssDNA, whose sequence corresponded with 169th to 198th position of klk1b26 mRNA) did not show the inhibitory effect.

**Conclusions:**

The small non-coding RNA, most probably miRNA, specifically expressed in female mouse SMGs interfered with klk1b26 protein synthesis in the *in vitro *translation system. Therefore sexual dimorphism observed in klk1b26 expression in mouse SMGs is due at least in part to the female-specific small non-coding RNA in SMGs.

## Background

It is known that the submandibular gland (SMG) of the male mouse contains many important bioactive substances such as nerve growth factor (NGF), epidermal growth factor (EGF), renin, and members of the kallikrein (klk) gene family [1-3]. Among these kallikrein isozymes, klk1, klk9, klk22, and klk1b26 have been identified as true tissue kallikrein, EGF-binding protein, β-NGF endopeptidase, and prorenin-converting enzyme, respectively [4,5]. One of the most prominent characteristics of these bioactive substances in mouse SMGs is the sex difference in their content [4-8]. The expression of klk9, klk22, and klk1b26 proteins in the ICR mouse is androgen responsive: the contents of these kallikreins are much higher in the male SMG than in the female one, castration decreases their contents to levels similar to the female ones, and 5α-dihydrotestosterone administration to females or castrated animals increases the content of these proteins [4-6]. This sex difference is thought to be due to increased mRNA synthesis stimulated by androgen [9,10]. However, we still have little information about the post-transcriptional regulation of klk1b26 protein in female mouse SMGs. The difference between klk1b26 mRNA levels in male and female mouse SMGs detected by reverse transcription (RT)-PCR does not seem large enough to fully account for the difference in klk1b26 protein levels between male and female SMGs. Furthermore, we observed that a small non-coding RNA fraction prepared from female mouse SMGs, but not male ones, inhibited PCR product formation for klk1b26 mRNA in RT-PCR [11].

For more than a decade, numerous studies have accumulated showing that small non-coding RNAs typically such as microRNAs (miRNAs), played important roles in controlling gene expression *via *post-transcriptional regulation. MicroRNAs are RNA molecules, 21 to 23 nucleotides in length, involved in post-transcriptional gene silencing mediated by the RNA interference pathway [12-21]. They are transcribed by RNA polymerase II as part of capped and polyadenylated primary transcripts (pri-miRNAs) that can be either protein coding or non-coding. The primary transcript is cleaved by the Drosha ribonuclease III enzyme to produce an approximately 70-nucleotide stem-loop precursor miRNA (pre-miRNA), which is further cleaved by the cytoplasmic Dicer ribonuclease to generate the sense miRNA and antisense miRNA star (miRNA*) products. The mature miRNAs (either sense or antisense sequences, or occasionally both sequences) play important roles in the regulation of translation and degradation of target mRNAs through base pairing to partially complementary sites in the 3'-untranslated regions (3'-UTR) of the mRNAs [17]. Post-translational regulation of protein expression by mature miRNA that interacts with its target mRNA at sites other than the 3'-UTR, for example at the coding region, has also been reported [21].

In the present study, we examined the effects of miRNA preparations from male and female SMGs on klk1b26 protein synthesis. We found that the female miRNA preparation, but not the male one, interfered with *in vitro *klk1b26 translation in a reticulocyte lysate system. The female miRNA preparation also inhibited PCR product formation only when primer pairs targeting 5'-terminal regions of the klk1b26 mRNA were used. Furthermore, synthetic small RNAs (20 to 23 nucleotides long) having partial homology with the complementary sequence at the 5'-terminal region of klk1b26 mRNA showed similar effects as the female miRNA preparation on the klk1b26 translation *in vitro*. The interfering effect of the female miRNA preparation on klk1b26 translation was lost by incubating it with a 30-nucleotide-long single-strand DNA whose sequence corresponded to the 5'-terminal region of klk1b26 mRNA sequence prior to the *in vitro *translation reaction, while the single-strand DNA itself had no effect on the klk1b26 translation. These results suggest that small non-coding RNAs such as miRNA specifically expressed in female SMGs are involved, at least in part, in the sexual dimorphism of klk1b26 expression in the mouse SMG.

## Methods

### Animals and treatments

ICR mice were purchased from Tokyo Laboratory Animal Science Co. Ltd. (Tokyo, Japan). All experiments were carried out in accordance with the guidelines for animal experiments of Meikai University. All animals were killed at 12 weeks of age for all of the experiments. Castration was carried out under ether anesthesia 4 weeks before hormone treatments. 5α-Dihydrotestosterone (DHT) was suspended in sesame oil. All hormone injections were made subcutaneously six times with a 1-day interval at a dose of 20 mg/kg DHT. At 1 day after the last hormone injection, the animals were killed by cervical dislocation and their SMGs were removed [6].

### RNA preparation

Total RNA and miRNA were prepared with a Micro-to-Midi Total RNA Purification System and a PureLink miRNA Isolation Kit, respectively (Invitrogen Life Technologies, Carlsbad, CA, USA) according to the manufacturer's instructions [22-24]. One sample of total RNA or of miRNA preparation was prepared from the SMGs of one mouse. Messenger RNA was prepared with a MicroPoly(A)Purist Small Scale mRNA Purification Kit (Ambion, Austin, TX, USA).

### Exo-mRNA fraction preparation

The exo-mRNA fraction was prepared by removing poly(A)-containing RNA from the total RNA by using oligo(dT) cellulose from a MicroPoly(A)Purist Small Scale mRNA Purification Kit (Ambion, no. AM1919). In brief, total RNAs prepared from SMGs of three mice were combined and mixed with a premeasured aliquot of oligo(dT) cellulose in binding solution, and the mixture was incubated to allow hybridization between the poly(A) sequences and oligo(dT) cellulose. The oligo(dT) cellulose was transferred to a spin column and eluted with non-binding solution. The exo-mRNA fraction in the flow-through solution was concentrated by using a Micro-to-Midi Total RNA Purification System Kit.

### RT-PCR

Reverse transcriptase reactions were carried out with a SuperScript First-Strand Synthesis System for RT-PCR (Invitrogen Life Technologies). In all experiments, the first-strand cDNAs were synthesized by using the oligo(dT) primer and then subjected to RNase H digestion [22-24]. PCR primers for mouse klk1b26 mRNA (GenBank: NM 010644) were designed based on the cDNA sequences registered in the Query Gene Bank Database, National Center for Biotechnology. Total RNAs (200 ng each) from tissues were used for the RT-PCR with Super Mix High Fidelity DNA Polymerase (Invitrogen Life Technologies), and PCR was performed with a Gene Amp 9600 PCR System (Perkin Elmer, Foster City, CA, USA) according to the following schedule: denaturation, annealing, and elongation at 94°C for 30 s, 55°C for 30 s, and 68°C for 60 s, respectively, for 25 cycles. Amplified PCR products were separated electrophoretically in agarose gels. Sequences of PCR products were confirmed to be identical to the corresponding positions of the cDNA sequences.

Primers, small RNAs and single-strand DNAs were synthesized by FASMAC Co., Ltd (Kanagawa, Japan) and their sequences were listed in Table 1.

**Table 1:** Nucleotide sequence of primers and oligonucleotides used in the experiments

Name of primers/oligonucleotides	Sequence (5'-3')	Corresponding position on klk1b26 mRNA (NM 010644)
DNA:		
F1 (forward primer)	agctccaagctcactgcctg	1-20
F11 (forward primer)	tcactgcctgcagttcctg	11-30
F15 (forward primer)	tgcctgcagttcctggacac	15-34
F21 (forward primer)	cagttcctggacacctgtta	21-40
F24 (forward primer)	ttcctggacacctgttacc	24-42
F40 (forward primer)	accatgtggttcctgatcct	40-59
F100 (forward primer)	cctctccagtctcgggtggt	100-119
F169 (forward primer)	tactaccaaaaggaacacattt	169-190
R552 (reverse primer)	atcatctgacttttgccatc	533-552 (complementary)
R609 (reverse primer)	taggtagactttggcacagtctc	586-609 (complementary)
[15th-44th]ssDNA	tgcctgcagttcctggacacctgttaccat	15-44
[169th-198th]ssDNA	tactaccaaaaggaacacatttgtgggggt	169-198
RNA:		
hsa-miR-325	ccuaguagguguccaguaagugu	21-40 (partly complementary)
miR-1497a	uugaagaacugcaggugguggau	12-28 (partly complementary)
asR-F21	uaacagguguccaggaacug	21-40 (complementary)

klk1b26 = kallikrein 1b26; ssDNA = single-stranded DNA.

### Sequence analysis of PCR products

For the sequence analysis, PCR products were reamplified with their respective primers in the presence of a dye terminator (BigDye Terminator Cycle Sequencing FS Ready Reaction Kit, no. 4303573; Perkin Elmer, Branchburg, NJ, USA) [22-24]. The DNA sequences were analyzed with an ABI PRISM 310 Genetic Analyzer (Perkin Elmer, Foster City, CA, USA).

### Sequence analysis of klk1b26 mRNA 5'-terminus by rapid amplification of cDNA ends (RACE)

The klk1b26 mRNA 5'-terminus was analyzed by use of a 5' RACE System, Version 2.0 (Invitrogen Life Technologies) according to the manufacturer's instructions. First-strand DNAs were synthesized from total RNAs in mouse SMGs by using a gene-specific reverse primer, 5'-taggtagactttggcacagttctc-3' (nucleotide positions 586 to 609, R609). A homopolymeric tail was added to the 3'-end of the cDNA by using terminal dioxynucleotidyl transferase (TdT) and dCTP. PCR amplification was accomplished by using Taq DNA polymerase with a primer pair of a nested gene-specific reverse primer (5'-tttggcacagttctcattgg-3': nucleotide positions 581 to 600, R600) and abridged anchor primer. The PCR product was reamplified with a primer pair of a nested gene-specific reverse primer (5'-atcatctggcttttgccatc-3' nucleotide positions 533 to 552, R552) and abridged universal anchor primer, resulting in the RACE product. The sequences of the RACE products were analyzed as described in the Sequence analysis of PCR products section below [22-24].

### SMG extract preparation for RNA degrading activity

SMGs were homogenized in buffer comprising 20 mM 4-(2-hydroxyethyl)-1-piperazineethanesulfonic acid (Hepes) (pH 7.3), 110 mM potassium acetate, 1 mM magnesium acetate, 2 mM CaCl_2 _and 1 mM dithiothreitol with a Teflon pestle glass homogenizer (Potter Elvehjem type). The homogenate was centrifuged at 29,700 *g *for 30 min, and the resulting supernatant was used for experiments.

### Preparation of anti-klk1b26 antibody and immunoprecipitation of klk1b26 protein

The klk1b26 protein was purified as described previously [5]: in brief, male mouse SMGs were homogenized in 20 mM Tris/HCl (pH 7.5) with a Teflon pestle glass homogenizer (Potter Elvehjem type). The homogenate was centrifuged at 105,000 *g *for 60 min, and the resulting supernatant was adjusted to pH at 4.5 with acetic acid. After centrifugation, the soluble fraction was neutralized with KOH, and then treated with 40% to 60% acetone. The precipitated protein was applied to isoelectric fractionation by the method of Vesterberg and Svensson [25]. The klk1b26 protein was isolated at pI 9.9 [5]. Anti-klk1b26 antiserum was obtained by immunizing a rabbit with the purified pI 9.9 protein as immunogen.

The antibody, preconjugated to protein G-beads, was incubated with the reticulocyte lysate mixture for 2 h in phosphate-buffered saline (PBS)/1% ovalbumin containing 1/100 × protease inhibitor cocktail (no. 535140; Calbiochem, La Jolla, CA, USA) for immunoreaction with the protein [8,26]. The immunoprecipitated protein was applied to non-reducing SDS-PAGE (16% polyacrylamide gel). Autoradiography was conducted on the dried gels.

### *In vitro *translations of klk1b26 protein and glyceraldehyde-3-phosphate dehydrogenase (GAPDH) with reticulocyte lysate

**Figure 4:**
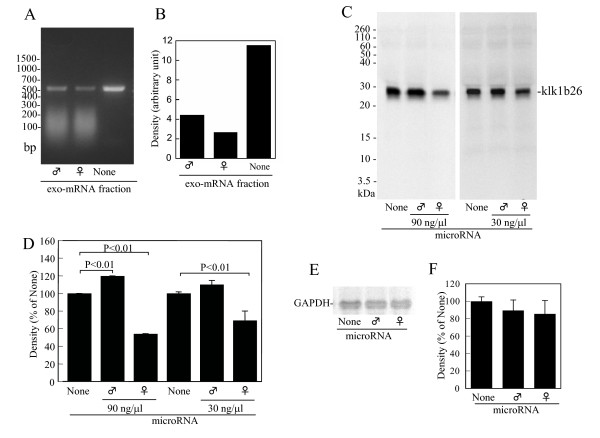
**Effects of exo-mRNA fraction on reverse transcription (RT)-PCR for kallikrein 1b26 (klk1b26) mRNA and inhibition of klk1b26 translation by miRNA preparation from female submandibular glands (SMGs)**. **(A) **Effect of exo-mRNA fraction on RT-PCR with a primer pair targeting the 5'-terminal region of klk1b26 mRNA. RT-PCR was carried out with the primer pair F21 forward and R552 reverse in the presence or absence of the exo-mRNA fraction (100 ng/μl) from male or female SMGs. **(B) **Quantitative determination of density of klk1b26 PCR products in Figure 4A was measured by computer-assisted image analysis (NIH image; http://rsbweb.nih.gov/nih-image/). Values are averages of the duplicate assay. **(C, D) **Inhibition of klk1b26 translation *in vitro *by miRNA preparation from female mouse SMGs. After the preincubation of mRNAs purified from male SMGs with miRNA preparation from male or female SMGs, the *in vitro *translation was performed and klk1b26 protein synthesized was analyzed as described in Methods. Representative results are shown. Similar results were obtained from three independent experiments (C). Quantitative determination of density of the [^35^S]klk1b26 protein band. Values represent the mean ± SD (n = 3) of the relative density (D). **(E) **Representative autoradiograms of [^35^S]methionine-labeled glyceraldehyde-3-phosphate dehydrogenase (GAPDH) protein on the SDS-PAGE gels. *In vitro *translation was performed as described in Methods. GAPDH protein was immunoprecipitated with anti-GAPDH antibody. **(F) **Quantitative determination of density of the [^35^S]klk1b26 protein band. Values are the mean ± SD (n = 3) of the relative density.

Protein synthesis by *in vitro *translation with a reticulocyte lysate system was carried out by using a Retic Lysate IVT for *in vitro *translation of mRNA (Ambion). Messenger RNA (500 ng) purified from male SMGs in an 11.2 μl translation mixture containing 1.4 μl of [^35^S]methionine (no. NEG709A; Perkin Elmer, Waltham, MA, USA) was mixed with 23.8 μl of reticulocyte lysate and incubated for 2 h at 30°C. When the effects of miRNA preparations from SMGs were examined, the mRNA (500 ng) purified from male SMGs was preincubated with miRNA preparations from male or female SMGs (final concentration of 90 or 30 ng/ml each for Figure 4C, D, and of 90 ng/ml each for Figure 4E, F) for 30 min at 4°C in a 11.2 μl translation mixture containing [^35^S]methionine (no. NEG709A; Perkin Elmer, Waltham, MA, USA). When the effects of miRNAs or antisense RNA (asR-F21) were examined, the mRNA preparation was preincubated with either of the synthetic miRNAs or asR-F21 in the translation mixture prior to mixing with the reticulocyte lysate. The translation reaction was stopped by adding 3.5 μl of Ambion RNase Cocktail Enzyme Mix (no. AM2286). Proteins synthesized in the *in vitro *system were mixed with anti-klk1b26 antibody or anti-GAPDH antibody (sc-25778; Santa Cruz Biotechnology, Santa Cruz, CA, USA) preconjugated to protein G-beads (no. 22851; Pierce Biotechnology, Rockford, IL, USA) and incubated for 2 h in PBS/1% ovalbumin containing 1/100 × protease inhibitor cocktail (no. 535140, Calbiochem). The immunoprecipitated protein was subjected to non-reducing SDS-PAGE (16% polyacrylamide gel). Autoradiography was conducted on the dried gels. Quantitative determination of density of the [^35^S]klk1b26 protein and [^35^S]GAPDH protein bands was performed by computer assisted image analysis of the autoradiograms.

### Preincubation of the female miRNA preparation with single-strand DNA for hybridization

MicroRNA preparations (3.5 μg) from female mouse SMG were incubated with 40 pmol chemically synthesized single-strand DNA, [15th-44th]ssDNA or [169th-198th]ssDNA, in 4 μl incubation mixtures containing 80 mM CH_3_COOK, 0.5 mM MgCl_2 _and 10 mM creatine phosphate (pH 7.4) at 80°C for 1 min and then slowly cooled down to 4°C in 10 min. The sequences of the single-strand DNAs are described in Table 1.

### Western blot analysis

SMGs were homogenized with a Teflon pestle glass homogenizer (Potter Elvehjem type) in 0.25 M sucrose containing 1/100 volume Proteinase Inhibitor Cocktail Set III (no. 535140; Calbiochem, Darmstadt, Germany). The homogenate was centrifuged at 29,700 *g *for 30 min, the resulting supernatant was subjected to non-reducing SDS-PAGE (16%), and the separated proteins were electrophoretically transferred onto polyvinylidene difluoride (PVDF) membrane filters. The filters were then stained immunochemically. In brief, filters were blocked with 5% skimmed milk in 10 mM Tris/HCl, pH 7.4, containing 150 mM NaCl and 0.05% Tween 20 (T-TBS) at room temperature for 45 min and then incubated for 1 h with the anti-klk1b26 antibody in T-TBS containing 5% skimmed milk. After having been washed with T-TBS, these filters were incubated for 1 h with anti-rabbit IgG goat serum conjugated with horseradish peroxidase (SC-2054; Santa Cruz Biotechnology). The filters were then washed, and the signal was detected with ECL Plus western blotting detection reagents (RPN 2132; Amersham, Buckinghamshire, England) [22-24].

### Statistical analysis

Statistical analyses were performed with a computer package (Microsoft Excel; Microsoft, Redmond, WA, USA). Data were presented as means ± SD. Levels of statistical significance between means were calculated by the two-tailed Student's t test.

## Results

### klk1b26 protein and RT-PCR of klk1b26 mRNA in mouse submandibular glands (SMGs)

**Figure 1:**
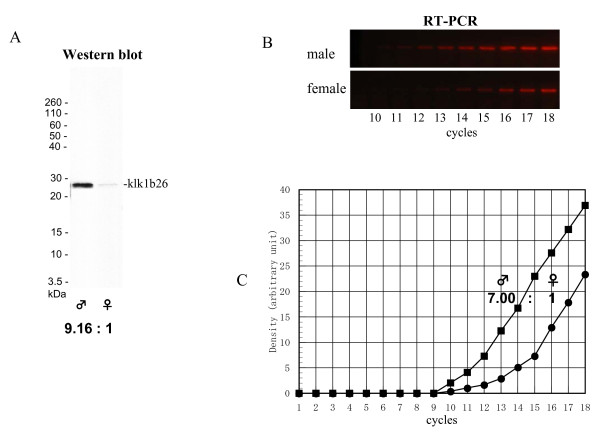
**Kallikrein 1b26 (klk1b26) protein and its mRNA levels in submandibular glands (SMGs) of male and female mice**. **(A) **Western blot analysis of klk1b26 proteins in SMGs. One sample of SMG protein extract was prepared from one mouse (two SMGs). The protein extracts from male and female SMGs (10 ng protein each) were subjected to SDS-PAGE (16% non-reducing gel), and blotted onto a polyvinylidene difluoride (PVDF) filter. The klk1b26 protein was detected with anti-klk1b26 polyclonal antibody. Quantitative determination of the klk1b26 signals was made by computer-assisted image analysis (NIH image; http://rsbweb.nih.gov/nih-image/). The klk1b26 protein ratio of male SMG to female SMG was estimated to be 9.16 ± 1.64 (mean ± SD; n = 3 mice). **(B) **Quantitative reverse transcription (RT)-PCR analysis of klk1b26 mRNA in SMGs. First-strand DNAs were prepared with 200 ng of total RNAs from male and female SMGs by using oligo(dT) primer. PCR was performed with primer pairs (F169/R552) targeting the middle region (169th to 552nd) of klk1b26 mRNA. The PCR conditions used were as follows: 94°C for 30 s, 55°C for 30 s, and 68°C for 120 s for denaturation, annealing, and elongation, respectively. **(C) **Quantitative determination of density of klk1b26 PCR products was made by computer-assisted image analysis, NIH image. The klk1b26 mRNA level of male SMG was estimated to be sevenfold higher than that of female SMG from the results of duplicate experiments.

One of the most prominent characteristics of kallikrein isozymes, including klk1b26 in mouse SMGs, is the sex difference in their content. We observed that klk1b26 enzyme activity based on extracted protein weight was approximately 9-fold higher in the male SMG than in the female [5]. From the western blotting and computer-assisted image analysis of klk1b26, the protein level (based on extracted protein weight) in male mouse SMGs was calculated to be 9.2-fold higher than that in female mouse SMGs (Figure 1A), indicating that the difference in the enzyme activities between male and female SMGs was simply due to the difference in the amount of klk1b26 protein between the male and female glands. This sex difference had been thought to be due to increased mRNA synthesis caused by androgen. We also examined klk1b26 mRNA levels by quantitative RT-PCR with a primer pairs targeting its middle region (169th to 552nd nucleotide region in klk1b26 mRNA of 873 nucleotides in length: NM 010644). The mRNA level in male SMGs was estimated to be sevenfold higher than that in female SMGs based on total RNA weight (Figure 1B, C). However, amounts of proteins and total RNAs (based on the tissue wet weight) extracted from male and female SMGs were considerably different from each other. Total proteins extracted from male and female SMGs by ordinary procedures used for various tissues were typically 105 ± 4 mg/g wet tissue and 56.5 ± 5.2 mg/g wet tissue (mean ± SD; n = 4 each sex), respectively; total RNAs usually extracted from male and female SMGs were 1,360 ± 232 ng/mg wet tissue and 1,932 ± 294 ng/mg wet tissue (mean ± SD; n = 4 each sex), respectively. When the klk1b26 protein and its mRNA in male and female SMGs were compared based on the SMG wet weights, males had 17-fold more klk1b26 protein than did the females, whereas the was fivefold more klk1b26 mRNA in males than females. If klk1b26 protein synthesis in mouse SMGs was proportional to the klk1b26 mRNA level, the klk1b26 protein content in female SMGs would be expected to be 20% of that of the same wet weight of male SMGs. However, the protein content per tissue wet weight in the female glands was estimated to be less than 6% of that in the male glands, suggesting a presence of some unknown factor(s) or mechanism(s), most probably post-transcriptional, down-regulating the klk1b26 expression in the female SMG.

### PCR product formation with F21 and F169 forward primers for klk1b26 mRNA from SMG and effects of castration and testosterone administration on the PCR signal strength

**Figure 2:**
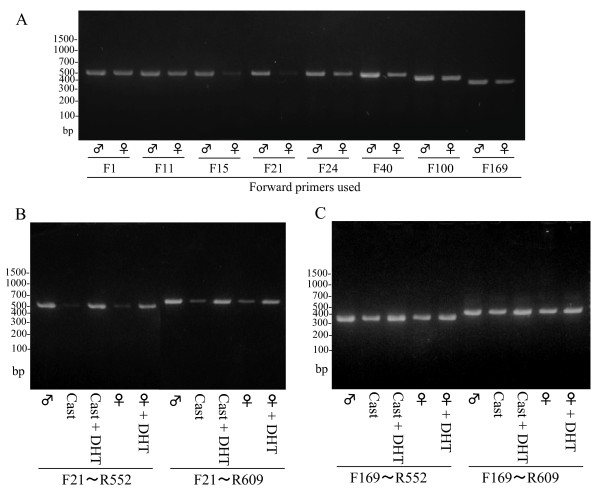
**Reverse transcription (RT)-PCR for kallikrein 1b26 (klk1b26) mRNA by using various forward primers targeting the 5'-terminal region and middle region of the mRNA**. **(A) **Positions of forward primers indicated the ordinal numbers of the nucleotides in the klk1b26 mRNA sequence (NM 010644) from its 5'-terminal end. Each forward primer was used in combination with the R552 reverse primer. Sequences of those primers are described in Table 1. **(B, C) **Total RNAs of ICR mouse submandibular glands (SMGs) were prepared from the following sources: male, castrated male (Cast), Cast + 5α-dihydrotestosterone (DHT), female, and female + DHT. **(B) **RT-PCR with primer pairs targeting the 5'-terminal region of klk1b26 mRNA was carried out by using either the primer pairs of F21 forward primer and R552 reverse primer or F21 forward primer and R609 reverse primer. **(C) **RT-PCR was carried out by using the F169 forward primer and R552 reverse primer or the F169 forward primer and R609 reverse primer targeting the middle region of klk1b26 mRNA. In (A-C), representative results are shown; similar results were obtained in three independent experiments.

**Figure 3:**
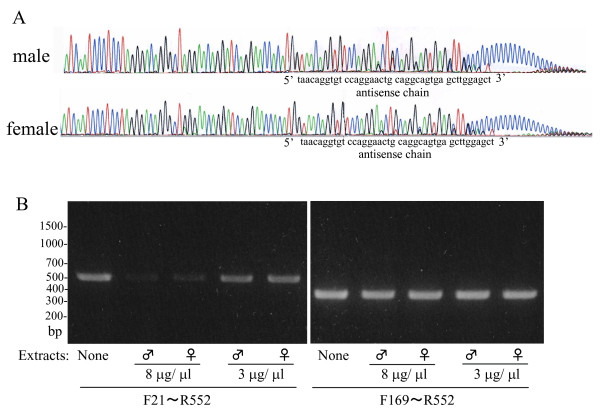
**5' Rapid amplification of cDNA ends (RACE) analysis of kallikrein 1b26 (klk1b26) mRNAs from male and female submandibular glands (SMGs) and effect of incubation with SMG extracts on the mRNA**. **(A) **Antisense chains of 5' RACE products were prepared and analyzed as described in Methods. Each signal is indicated by color: green = A; red, = T; black = G; blue = C. **(B) **Degradation of klk1b26 mRNA by incubation with the SMG extracts. Total RNA from male SMGs (21.5 μg) was incubated at 37°C for 30 min with an extract prepared from male or female mouse SMGs as described in Methods. After the incubation the total RNA was again purified with the Micro-to-Midi Total RNA Purification System (Invitrogen) and was eluted to 20 μl, quantitatively. First-strand DNAs were prepared with 280 ng of the total RNAs thus treated with SMG extracts and reverse transcription (RT)-PCR was carried out by using primer pairs F21/R552 or F169/R552. Representative results are shown. Similar results were obtained in three independent experiments. Quantitative determination of density of the PCR products was made by computer-assisted image analysis (NIH image; http://rsbweb.nih.gov/nih-image/). Relative densities of the PCR signals with total RNAs treated by 8 μg/μl each of male SMG extract and female SMG extract were estimated to be 7.36 ± 0.40 and 7.44 ± 0.51, respectively (mean ± SD; n = 3 each). There was no significant difference in the degrading activities for the mRNA between the male and female SMG extracts.

In order to reexamine the efficiency of primers we carried out RT-PCR for klk1b26 mRNA of male and female mouse SMGs with various sets of primer pairs targeting various regions of the mRNA. As shown in Figure 2A, primer pairs that amplified the middle regions of the mRNA failed to reveal a clear sex difference in their PCR products. Only two primer pairs, F15/R552 and F21/R552, both of which targeted near the 5'-terminal region of the mRNA, exhibited a remarkable difference in the PCR signals for the mRNA between male and female SMGs; the F1/R552 pair, which targeted the 5'-terminal, did not show a clear difference. The difference in the signal strength between male and female was greater with the F21/R552 pair than with the F15/R552 one. Both the F21/R552 and F15/R552 primer pairs produced substantial PCR products when the first-strand cDNA from male total RNA was used, indicating that the lesser signal intensity detected with the female RNA preparation was not due to an inefficiency of these primers for the PCR (Figure 2A). These results suggest a possible modification of klk1b26 mRNA in female SMGs at a position between the 15th and 40th nucleotide. This possibility was examined later (Figure 3).

Since a clear sex difference in the PCR signals for klk1b26 mRNA levels in SMGs was detected with the F21/R552 or F15/R552 primer pair, we examined whether the signal strength was affected by androgen. The first-strand cDNAs were prepared by using oligo(dT) primer and total RNA from SMGs of experimental animals. Both primer pairs having the F21 forward primer, F21/R552 and F21/R609, gave a clear sex difference in the PCR signals; the effect of androgen, that is, castration or DHT administration to females or castrated mice, on the signal strength was clearly observed with these primer pairs (Figure 2B). However, when PCR was performed with the F169 forward primer (primer pairs of F169/R552 and F169/R609), a much smaller difference in the PCR signals for the mRNA levels between male and female SMGs was found (Figure 2C). Also, there were only small differences in the PCR signals for klk1b26 mRNA in SMGs between males and castrated males or those given DHT, and in SMGs between females and DHT-administered females.

### Nucleotide sequences at the 5'-terminal regions of klk1b26 mRNAs from male and female SMGs and effect of incubation with SMG extracts on the mRNA

We then examined the nucleotide sequence of klk1b26 mRNAs from male and female SMGs by use of the 5' RACE method, since the results of experiments shown in Figures 1 and 2 suggested the possibility that klk1b26 mRNA in SMGs of females and castrated mice had undergone some post-transcriptional modification near its 5'-terminal region, somewhere between the 15th and 40th nucleotide. However, we unexpectedly obtained the same nucleotide sequences for both the male and female mRNAs from their 5'-terminal to 40th nucleotide, 5'-agctccaagctcactgcctgcagttcctggacacctgtta-3', which coincided with the corresponding part of the registered klk1b26 mRNA sequence (GenBank: NM 010644, Figure 3A), indicating no modification in the 5'-terminal region of klk1b26 mRNA in female SMGs.

We also examined protein extracts from male and female SMGs for any activity modifying klk1b26 mRNA (Figure 3B). *In vitro *incubation of the total RNA prepared from male SMGs with cell-free extracts of either male or female SMGs decreased the PCR signals obtained with the F21/R552 primer pair. However, the PCR signals with the F169/R552 primer pair were not so strongly affected by the SMG extracts from male or female, suggesting that the 57-terminal region of klk1b26 mRNA might be more fragile or susceptive to RNA degradation than its middle region. However, a significant difference in mRNA degrading activities was not detected between the male and female SMG extracts.

### Effects of miRNA preparations from mouse SMGs on *in vitro *translation of klk1b26 mRNA

It is unlikely from the results shown in Figure 3 that klk1b26 mRNA in female SMGs underwent post-transcriptional modification at detectable levels. We then examined the presence of some substance(s) in the total RNA preparation from female SMGs, which inhibited the PCR reaction performed specifically with the F15 or F21 forward primers. We prepared an 'exo-mRNA fraction' from total RNA preparations made from male and female SMGs by removing RNAs with the poly(A) sequence, and tested its effect on RT-PCR. The female 'exo-mRNA fraction' seemed to more strongly inhibit RT-PCR for klk1b26 mRNA performed with the F21/R552 primer pair than the male 'exo-mRNA fraction' (Figure 4A, B). Neither 'exo-mRNA fraction' from male or female SMGs gave detectable effects on the RT-PCR performed with the F169/R552 primer pair (data not shown).

The interfering substance in the female total RNA preparations somehow acted in a sequence-specific manner and was assumed to be a form of small non-coding RNA contaminating in the total RNA preparations. Since small non-coding RNAs such as miRNA are known to play important roles in controlling gene expression, we prepared miRNA fractions from male and female mouse SMGs and examined their effects on klk1b26 protein synthesis. In the *in vitro *translation system composed of reticulocyte lysate with mRNAs purified from male SMGs, the female miRNA preparation dose dependently inhibited the synthesis of klk1b26 protein (Figure 4C, D): the autoradiogram signal of the klk1b26 protein in the presence of the female miRNA decreased to 50% to 70% of that of the protein in the absence of the miRNA preparation. However, the male miRNA preparation did not have such an inhibitory effect on the *in vitro *klk1b26 translation (Figure 4C, D). In the control experiments, a slight decrease in GAPDH translation was sometimes observed by the addition of the miRNA preparations. However, the effects of both the miRNA preparations from male SMGs and female ones on the synthesis of GAPDH protein synthesis were not at significant levels (Figure 4E, F). Additionally, there was no significant difference in the effects of male and female miRNA preparations on the GAPDH translation.

### Effect of miRNA preparation on RT-PCR of klk1b26 mRNA

**Figure 5:**
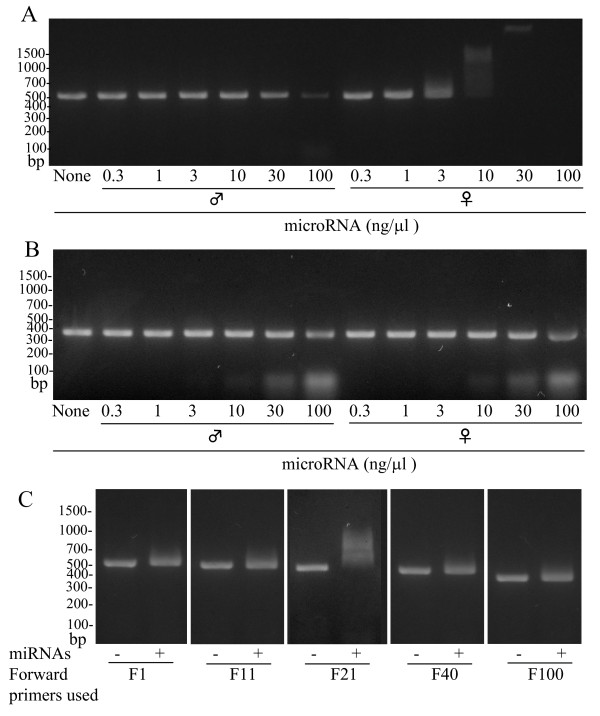
**Effect of miRNA preparations on reverse transcription (RT)-PCR for kallikrein 1b26 (klk1b26) mRNA**. **(A,B) **Effect of miRNA preparation on the PCR product formation. RT-PCR was performed with the F21/R552 primer pair targeting the 5'-terminal region of klk1b26 mRNA (A) or with the F169/R552 primer pair targeting the middle region of the mRNA (B) in the presence of the indicated concentrations of miRNA prepared from either male or female submandibular glands (SMGs). Representative results are shown, and similar results were obtained in three independent experiments. **(C) **Effect of female miRNA preparation on the PCR product formation using various forward primers targeting the 5'-terminal region. PCR was carried out with each forward primer in combination with the R552 reverse primer in the absence (-) or presence (+) of miRNAs prepared from female SMGs (10 ng/μl). Sequences of the primers used are indicated in Table 1. Representative results are shown. Similar results were obtained in three independent experiments.

Since the miRNA preparation from female SMGs inhibited the klk1b26 translation (Figure 4C, D), we also examined its effects on the RT-PCR of klk1b26 mRNA. The miRNA preparation from female SMGs strongly inhibited RT-PCR with the F21/R552 primer pair, whereas that from male SMGs showed only a slight effect even when used in high amounts (Figure 5A): the female miRNA was approximately 30 times more effective than the male miRNA in inhibiting the PCR reaction. In contrast, PCR performed with the F169/R552 primer pair targeting the middle region of klk1b26 mRNA was scarcely affected by either the miRNA preparations from male SMGs or female ones (Figure 5B). The effects of the female miRNA preparation on the RT-PCR of klk1b26 mRNA were also obscured when performed with other forward primers, F1, F11, F40 and F100, used in combination with the R552 reverse primer (Figure 5C).

### Effects of androgen on miRNAs in mouse SMGs

**Figure 6:**
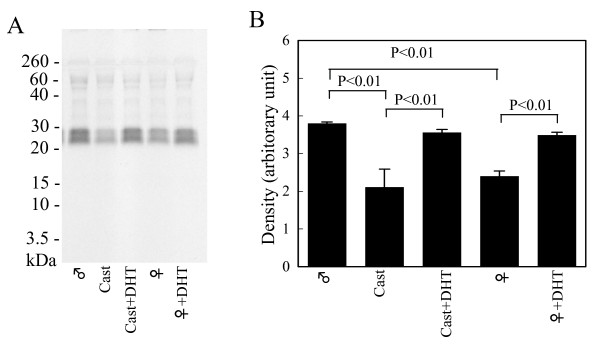
**Effects of castration and 5α-dihydrotestosterone (DHT) administration on the activity in the submandibular gland (SMG) miRNA preparation interfering kallikrein 1b26 (klk1b26) translation**. **(A) **MicroRNAs were prepared as described in Methods from SMGs of castrated mouse, DHT-administered castrated mouse and DHT-administered female mouse, respectively. One miRNA sample was prepared from SMGs from one mouse. The effects of these miRNA preparations (90 ng/μl each) on klk1b26 translation were analyzed as described in the legend for Figure 4C. Representative results are shown. Similar results were obtained in three independent experiments. **(B) **Quantitative determination of density of the [^35^S]klk1b26 protein band by computer-assisted image analysis of the autoradiograms. Values represent the mean ± SD (n = 3 animals) of the relative density.

Since the RT-PCR signal strength for klk1b26 mRNA of mouse SMG analyzed by using the F21/R552 or F15/R552 primer pairs was affected by androgen as shown in Figure 2B, we next examined effects of castration and DHT administration on the activity to inhibit klk1b26 translation in the miRNA preparations from SMGs. The miRNA preparation from castrated mice as well as normal female mice interfered with klk1b26 translation in the *in vitro *system, and DHT administration to castrated mice or female mice caused loss of the interfering activity in the miRNA preparations from their SMGs (Figure 6A, B). The results indicated that expression of the female-specific small non-coding RNA, probably miRNA, in SMGs that inhibited klk1b26 translation, was down-regulated by androgen.

### Effects of synthetic RNAs partially complementary with the 5'-region of klk1b26 mRNA on *in vitro *translation of klk1b26 protein

**Figure 7:**
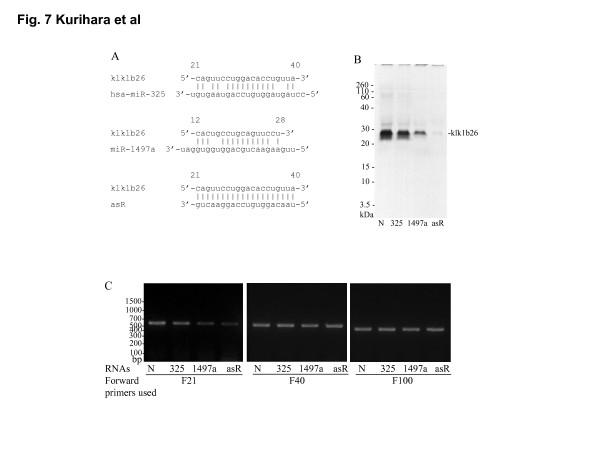
**Inhibition of *in vitro *kallikrein 1b26 (klk1b26) translation by synthetic miRNAs partially complementary with the 5'-region of klk1b26 mRNA**. **(A) **Sequences of miRNAs and their complementary sites in the 5'-region of klk1b26 mRNA. The numbers above the klk1b26 mRNA sequence indicate the nucleotide positions from the transcription start site (GenBank: NM 010644). **(B) **Inhibition of *in vitro *translation of klk1b26 by the miRNAs and antisense RNA of F21 forward primer (asR-F21). Messenger RNA (500 ng) purified from male submandibular glands (SMGs) was preincubated with or without miR-325, miR-1497a or asR-F21 (10 μM each) in a translation mixture. The mixture was then incubated with a reticulocyte lysate for *in vitro *protein synthesis, and [^35^S]methionine-labeled klk1b26 protein was analyzed by SDS-PAGE as described in Methods. N, 325, 1497a, and asR stand for the absence of synthetic RNA, miR-325, miR-1497a, and asR-F21, respectively. Representative results are shown. Similar results were obtained in three independent experiments. **(C) **Effects of synthetic miRNAs and antisense RNA of F21 forward primer (asR-F21) on reverse transcription (RT)-PCR for klk1b26 mRNA using various forward primers targeting the 5'-terminal region. Sequences of the primers are described in Table 1. PCR was carried out with each forward primer in combination with the R552 reverse primer in the absence or presence of the synthetic RNAs. N, 325, 1497a, and asR indicated the absence of synthetic RNA (3 μM), miR-325, miR-1497a, and asR-F21, respectively. Representative results are shown. Similar results were obtained in three independent experiments.

From the results of the experiments shown in Figures 4, 5, 6, it seemed likely that the RNA in the miRNA preparation from female SMGs that interfered with the klk1b26 translation *in vitro *also inhibited the RT-PCR of klk1b26 mRNA performed with the F21/R552 primer pair. Therefore, we first searched for small non-coding RNAs having some complementarity with the klk1b26 mRNA sequence at the position between the 15th and 44th nucleotide from the miRNA database. The miRBase search program [27,28] picked up miR-325 (5'-ccuaguagguguccaguaagugu-3', Figure 7A), whose sequence is conserved among *Homo sapiens *(hsa), *Macaca mulatta *(mml), and *Pongo pygmaeus *(ppy). In the mouse, a homologous sequence having two alternative nucleotides (mmu-miR-325*) was registered as a minor component [29,30]. Therefore we examined the effects of chemically synthesized miR-325 and antisense RNA of the F21 primer (asR-F21) on klk1b26 protein synthesis in the *in vitro *translation system. Both miR-325 and asR-F21 inhibited the *in vitro *translation of klk1b26 mRNA, with asR-F21 having the stronger effect (Figure 7B). Since the difference in PCR product formation for klk1b26 mRNA was also observed with F15 forward primer (Figure 2A), we examined the effects of the synthetic RNA sequence of miR-1497a (5'-uugaagaacugcaggugguggau-3'), which was expressed in *Oikopleura dioica *(odi), on *in vitro *klk1b26 translation; however its counterpart in mouse had not been registered in the miRBase database [31]. This miR-1497a also inhibited the synthesis of klk1b26 protein *in vitro *(Figure 7B).

When the effects of these synthetic RNAs on the RT-PCR for klk1b26 mRNA were tested using three sets of primer pairs as shown in Figure 7C, PCR product formation was inhibited by respective RNAs only when the F21 forward primer was employed but not with the F40 or F100 primers.

### Masking of the translation-interfering activity in the female miRNA preparation by specific single-strand DNA

**Figure 8:**
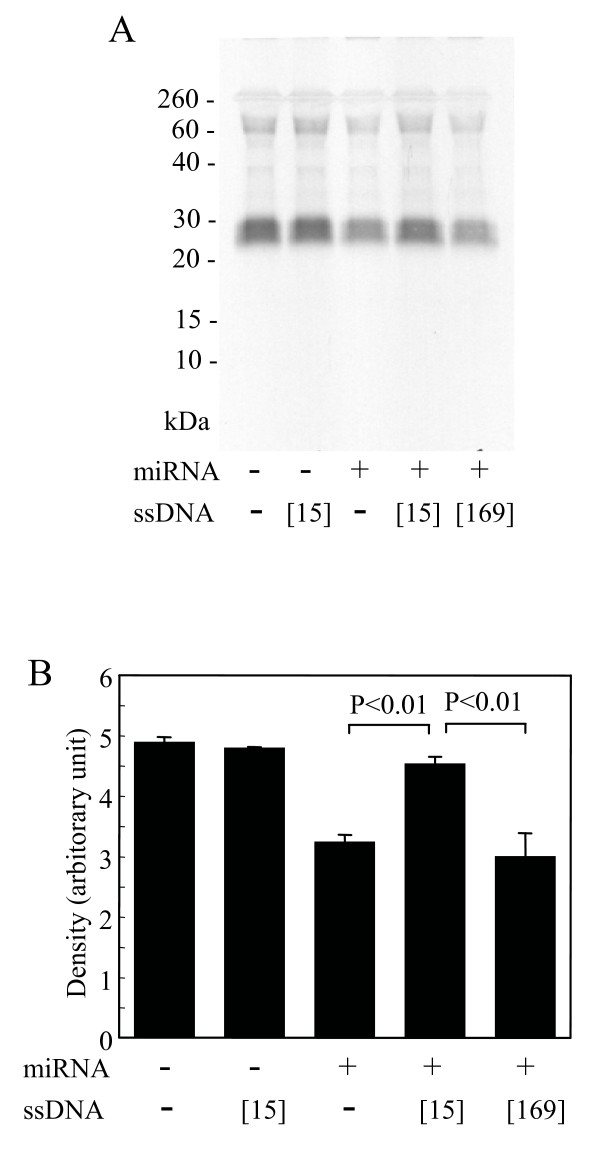
**Masking of the interfering effect of female miRNA preparation on kallikrein 1b26 (klk1b26) translation by specific single-strand DNA, [15th-44th]ssDNA**. **(A) **Masking of the activity in female miRNA preparation interfering klk1b26 translation by [15th-44th]ssDNA ([15]). The sequences of single-strand DNAs, [15th-44th]ssDNA and [169th-198th]ssDNA ([169]) (used as negative control), corresponded with the 15th to 44th and 169th to 198th nucleotide positions of klk1b26 mRNA, respectively, and are described in Table 1. Prior to the addition into the *in vitro *translation system, the female miRNA preparation was incubated with either of the single-strand DNAs. The *in vitro *translation reaction and the [^35^S]klk1b26 protein analysis were carried out as described in Methods. Representative results are shown. Similar results were obtained from three independent experiments. **(B) **Quantitative determination of density of the [^35^S]klk1b26 protein band. Values represent the mean ± SD (n = 3) of the relative density.

The results of the experiments shown in Figure 7A-C suggest that the klk1b26 translation inhibition was due to an interaction of the interfering RNA in the female miRNA preparation with klk1b26 mRNA at the 15th to 44th nucleotide position. Next, we examined whether a single-strand DNA, [15th-44th]ssDNA, whose sequence corresponded to the 15th to 44th residue of klk1b26 mRNA sequence, masked/neutralized the effect of the interfering RNA in the female miRNA preparation by forming a hybrid with it. The female miRNA preparation incubated with [15th-44th]ssDNA prior to the addition into the *in vitro *assay mixture lost its activity interfering with klk1b26 translation, while [15th-44th]ssDNA itself added into the assay mixture without the female miRNA preparation did not have a significant effect on klk1b26 translation (Figure 8A, B). Preincubation of the female miRNA preparation with another single-strand DNA [169th-198th]ssDNA whose sequence corresponded with the 169th to 198th nucleotide position of klk1b26 mRNA sequence failed to show the masking effect as [15th-44th]ssDNA did (Figure 8A, B). These results support the idea that the female-specific small non-coding RNA, probably miRNA, in the miRNA preparation from female mouse SMGs interfered with klk1b26 translation in the *in vitro *system by interacting with the specific position of klk1b26 mRNA, within the 15th to 44th nucleotide position from the 5'-terminal.

## Discussion

Mouse klk1b26, a prorenin-converting enzyme, is a member of the klk gene family [5] and is much more abundantly expressed in the SMGs of male mice than in females [6,7]. This sex difference in klk1b26 protein expression has been thought to be due to stimulated transcription of the klk1b26 gene in the SMG by androgen [9,10]. However, we found that when the klk1b26 protein and its mRNA levels in the male and female mouse SMGs were compared based on the wet weight of the glands, the mRNA levels in female SMGs were as much as 20% of that in male SMGs, while klk1b26 protein levels in the female glands were less than 6% of that in the male glands. Thus, we conceived the idea that there was an as yet unidentified mechanism, most probably post-transcriptional, down-regulating klk1b26 expression in the female SMG.

We reexamined klk1b26 mRNA levels in male and female SMGs by RT-PCR using various primer pairs targeting various regions of the mRNA, and found that the klk1b26 mRNA level in female total RNA preparations was estimated to be extremely low compared to that of male total RNA preparations only when the RT-PCR was performed with forward primers targeted near the 5'-terminal region of the mRNA (F15 and F21 primers, where the 5'-nucleotides corresponded to the 15th and 21st nucleotides of klk1b26 mRNA (GenBank: NM 010644), respectively) than when other forward primers targeting other regions of the mRNA were used (Figure 2). The inefficiency of the PCR product formation by F15 or F21 forward primers with the total RNA from female SMGs was not due to the modification of klk1b26 mRNA in female SMGs, because klk1b26 mRNAs from both male and female SMGs had the same nucleotide sequence according to the results of 5' RACE analysis (Figure 3A). The PCR products from male and female total RNA preparations with various primer pairs were subjected to DNA sequencing analysis and confirmed to be identical with the corresponding position of the klk1b26 mRNA sequence. Also, transcript variants for klk1b26 have not been reported/registered in the GenBank database. These results, therefore, suggest that female SMGs contained some inhibitory RNA molecules that interfered with the PCR process in a sequence-specific manner. The results of experiments with the 'exo-mRNA fraction' (Figure 4A, B) also supported this assumption.

Next, we examined the effects of miRNA from mouse SMGs on klk1b26 translation using the *in vitro *reticulocyte lysate system, because miRNAs arguably plays regulatory roles in gene expression and because such miRNA could be expected to interfere with the PCR reaction in a sequence-specific manner. The miRNA preparations from mouse SMGs prepared by using a PureLink miRNA Isolation Kit (Invitrogen Life Technologies) might contain small RNAs of < 200 nucleotides long other than miRNAs. The female miRNA preparation, but not the male one, inhibited the synthesis of klk1b26 protein in the *in vitro *translation system (Figure 4C, D). GAPDH translation in the same *in vitro *system was scarcely decreased by either the miRNA preparation from male or female, but the effects were not at significant levels. Differences between the effects of male and female miRNA preparations on GAPDH translation were not statistically significant. Since the klk1b26 translation interfering RNA was specifically observed in female mouse SMGs, the effects of castration and DHT administration to females or castrated mice were tested. The interfering RNA appeared in the SMG miRNA preparations from castrated mice and disappeared in mice who had undergone DHT administration (Figure 6A, B), indicating that expression of the interfering RNA was down-regulated by androgen.

The miRNA preparation from female SMGs also inhibited product formation in RT-PCR when performed with the primer pair (F21/R552) targeting the 5'-terminal region of klk1b26 mRNA, whereas the male miRNA preparation gave only a slight effect (Figure 5A). It should be noted that the inhibitory effect of the female miRNA preparation on the PCR product formation was clear when the F21 forward primer was used, but was not obvious with the F1, F11, F40, F100 (Figure 5C) or F169 forward primers (Figure 5B). These results implied that the length of RNA contained in the female miRNA preparation, which interacted with klk1b26 mRNA and interfered with klk1b26 translation, was not greater than 31 nucleotides long and that the site on the klk1b26 mRNA where the interfering RNA interacted was within 44 nucleotides from the start site of the mRNA (GenBank: NM 010644). We prepared small RNAs of 20 to 23 nucleotides long (chemically synthesized hsa-miR-325, miR-1497a, and asRNA in Figure 7A) which had partial complementarity with klk1b26 mRNA at its 5'-terminal region, and demonstrated that these small RNAs inhibited the *in vitro *translation of klk1b26 mRNA (Figure 7B). All of these synthetic small RNAs also inhibited the PCR reaction when the primer pair of F21/R552 was used. However, the inhibitory effect of the RNAs on PCR product formation was not detectable when the F40 or F100 forward primers were used in combination with the R552 reverse primer (Figure 7C). Considering the results shown in Figures 5 and 7 together with that shown in Figure 2A where the inhibition of PCR product formation was also observed with the F15 forward primer, it seemed plausible that the interfering RNA in the female miRNA preparation had partial complementarity with klk1b26 mRNA in its 15th to 44th nucleotide position, and its length was not greater than 25 nucleotides long. Therefore, we synthesized 30-nucleotide-long single-strand DNAs, [15th-44th]ssDNA and [169th-198th]ssDNA, of those sequences corresponding with the klk1b26 mRNA sequence at the 15th to 44th and 169th to 198th positions. Preincubation for hybridization of the female miRNA preparation with [15th-44th]ssDNA, but not with [169th-198th]ssDNA used as a negative control, masked/neutralized the activity of the female miRNA preparation in interfering with klk1b26 translation (Figure 8A, B), supporting our assumption.

Translational repression by miRNAs is thought to occur *via *several mechanisms: RNA-induced silencing complex (RISC)-mediated enhancement of deadenylation and decapping to destabilize the mRNA; sequestering of the mRNA into cytoplasmic bodies (GW-bodies or P-bodies); or disrupting the binding of translation factors to inhibit translation initiation or elongation [18,21]. When miRNA affects mRNA such as small interfering RNA (siRNA), miRNAs associated with argonaute proteins in the RISC repress protein expression by binding to regions of complementarity with the target mRNAs. The binding sites of miRNAs were typically located within the 3'-UTR of target mRNAs [15,18-21]. In this case, the seed sequence (second to eighth sequence from the 5'-end) of miRNA was thought to be very important and should be complementally matched with the target mRNA sequence [13,14]. However, miRNAs were also reported to repress translation by interacting with coding regions of target mRNAs [21]: mouse Nanog, Oct4 and Sox2 genes were demonstrated to have many naturally occurring miRNA targets in their amino acid coding sequence (CDS) and some of those targets were revealed to not contain the miRNA seed. Tay *et al*. [21] also reported that the miRNAs interacting at the CDS regions of their target mRNAs had only a limited effect on the mRNA levels but affected the corresponding proteins more substantially. We hypothesized that a kind of miRNA that was specifically expressed in female mouse SMGs interfered klk1b26 translation process contributing to sexual dimorphism of the klk1b26 protein in the mouse SMGs. It is plausible that such a miRNA, which has partial complementarity with the 5'-terminal region of klk1b26 mRNA, inhibits the klk1b26 protein synthesis by disturbing the binding of translation factors needed for translation initiation or elongation, though the effects of other kinds of small non-coding RNA(s) cannot be completely ruled out. In our preliminary experiments on miRNA profiling for male and female mouse SMGs by microarray assay, mmu-miR-325*, which is the mouse counterpart of primate miR-325, was not expressed in high enough levels to estimate whether it was one of the miRNAs expressed in female SMGs rather than in male SMGs. Though the sequence of mmu-miR-325* [29,30] has two alternative nucleotides compared with that of *Homo sapien*s (hsa) miR-325, whose sequence is conserved in *Macaca mulatta *(mml) and *Pongo pygmaeus *(ppy) [27,28], mmu-miR-325* is one of the plausible candidates. Additionally, there might be considerable numbers of miRs that have not yet been found and/or registered in miR database. Further analysis should be carried out for the identification of the miRNA(s) involved in the regulation of klk1b26 expression.

SMGs of male mice but not those of female mice produce various proteins with important biological activities [1-3]. Their expression is androgen dependent [1,6,7,9,10], and some of them are also responsive to thyroid hormone [6-10,32,33]. As implied in the present study, this sex difference observed in mouse SMGs is attributed, at least in part, to the miRNA(s) specifically expressed in the female SMG. More than 900 mature miRNAs are on file for human miRNAs in the miRNA database (miRBase release 16, September 2010; http://www.mirbase.org/) and over 60% of human protein-coding genes are thought to be targeted by miRNAs [34]. Since miRNAs play critical roles for the normal functioning of cells, dysregulation of miRNA is thought to be associated with many diseases. Many miRNAs are reported to have links with cancer [35], heart disease [36], and dysfunctions in the nervous system [37]. Among them are those for sex-specific diseases such as breast cancer [38,39], ovarian cancer [40,41], and prostate cancer [42]. The detailed mechanisms of the miRNA effects on those diseases largely remain to be elucidated. Furthermore, sexual dimorphism is observed not only in the diseases of reproduction-related organs but also in the prevalence and severity of many common diseases such as cardiovascular diseases, autoimmune diseases, asthma, Alzheimer's disease, and Parkinson's disease [43]. The sexual dimorphism observed in disease traits is thought to result from differential gene regulation in males and females, particularly with respect to sex hormone-responsive genes [43] including those that encode miRNAs.

Among the organs common in males and females, the mouse SMG is one that typically displays sexual dimorphism. It should, therefore, be noted that the mouse SMG is an important model system to investigate further the role and action mechanisms of sex-specific small non-coding RNAs including miRNAs and to uncover the regulatory mechanisms of such sex-specific RNA expression involving androgen and/or estrogen, and occasionally the combination of sex hormones with other hormones.

## Conclusions

Mouse klk1b26 protein is more abundant in male SMGs than in female ones; this difference had been thought to be due to only increased mRNA synthesis stimulated by androgen. In the present study a small non-coding RNA, most probably miRNA, specifically expressed in female mouse SMG interfered with klk1b26 protein synthesis in an *in vitro *translation system. Therefore, interference in the klk1b26 translation process by such a small RNA specifically expressed in females is at least in part involved in the sexual dimorphism of klk1b26 expression in the mouse SMG.

## Competing interests

The authors declare that they have no competing interests.

## Authors' contributions

KK treated the animals and process the tissue samples, and drafted the manuscript. Both KK and NN performed molecular biology, physiology and statistical analysis. AT conceived the study and participated in its design. All authors read and approved the final manuscript.

## Authors' information

KK works on the board of directors for the Journal of the Japan Salivary Gland Society.
